# *Porphyromonas gingivalis*‐derived outer membrane vesicles promote calcification of vascular smooth muscle cells through ERK1/2‐RUNX2

**DOI:** 10.1002/2211-5463.12151

**Published:** 2016-11-21

**Authors:** Wen Wei Yang, Bin Guo, Wen Yuan Jia, Yue Jia

**Affiliations:** ^1^ Department of StomatologyAviation General Hospital of China Medical UniversityBeijingChina; ^2^ Department of StomatologyThe General Hospital of Chinese People's LiberationBeijingChina; ^3^ Department of PeriodonticsWest China College of StomatologySichuan UniversityChengduChina

**Keywords:** extracellular‐regulated kinase, outer membrane vesicle, *Porphyromonas gingivalis*, runt‐related transcription factor 2, vascular calcification

## Abstract

The outer membrane vesicle (OMV) derived from *Porphyromonas gingivalis* plays an essential role in causing inflammation which, in turn, plays an important part in the pathogenesis of cardiovascular diseases such as atherosclerosis and thromboembolism. However, the contribution of oral bacteria to vascular calcification is yet to be determined. Here, we evaluated the effect of OMV on vascular smooth muscle cell (VSMC) calcification both *in vitro* and *ex vivo*. We established a reproducible *P. gingivalis* OMV‐induced differentiation and calcification model of VSMCs *in vitro*. The results indicate that OMV promotes VSMC calcification in a concentration‐dependent manner, modulating the expression of bone markers and SMC markers both on genes and proteins that are important for osteoblastic differentiation and mineralization of VSMCs. We also showed that the key osteogenic transcription factor, runt‐related transcription factor 2 (Runx2), which is affected by upstream extracellular‐regulated kinase (ERK) signaling, is a key regulator of OMV‐induced VSMC differentiation and calcification. Taken together, our research demonstrates that Runx2 is a crucial component of OMV‐induced calcification of VSMCs, and ERK signaling plays a vital role in mediating Runx2 up‐regulation and VSMC calcification.

**Abbreviations**
ALP: alkaline phosphatase
ColIA1: collagen typeIalpha 1
ERK: extracellular‐regulated kinase
OC: osteocalcin
OMV: outer membrane vesicle
Runx2: runt‐related transcription factor 2
SMA: smooth muscle‐specific α‐actin
VSMC: vascular smooth muscle cell

*Porphyromonas gingivalis* induces a local chronic host inflammatory response that ultimately leads to inflammatory alveolar bone resorption and tooth loss, which are the key features of periodontal disease 1. Evidence suggests that *P. gingivalis*‐mediated periodontal disease is the risk factor of cardiovascular disease because periodontal pathogenic bacteria have been detected in heart valve lesions, thrombus, and atheromatous plaque 2, 3, 4.

The outer membrane vesicle (OMV), released by *P. gingivalis* as extracellular membrane vesicles, retains major virulence including proteins, muramic acid, lipopolysaccharide (LPS), gingipain, fimbriae, and capsule 5, 6. OMV could therefore be internalized into host cells via a lipid raft‐dependent endocytic pathway 7, resulting in the activation of inflammatory pathways. OMV can be detected in the peripheral blood and cerebrospinal fluid in patients with severe bacterial infections 8, 9, which may be closely related to cardiovascular disease.

Vascular calcification, the accumulation of calcium deposits in the blood vessel, is a hallmark of atherosclerosis, causing angiosclerosis and reduced elasticity of the vessel 10. A variety of vascular cells, including vascular smooth muscle cells (VSMCs), myofibroblasts, vascular mesenchymal progenitors, and endothelial cells, could be involved in vascular calcification 11, 12. Many studies have now supported the theory that vascular calcification is an active cell‐driven process associated with osteoblast differentiation of vascular cells 13, 14, 15, which is manifested as the expression of numerous bone‐related molecules including alkaline phosphatase (ALP), collagen type I alpha 1 (ColIA1), osteocalcin (OC), and mineralized bone‐like structures 16, 17, 18. Runt‐related transcription factor 2 (Runx2) is a key transcription factor that regulates osteoblast 19 and chondrocyte 20 differentiation. Runx2 induces ALP activity and expression of bone‐related matrix proteins, including ColIA, OC, and osteopontin (OPN) 21, 22, 23, 24. Increased expression of Runx2 is associated with VSMC calcification *in vitro* 24, 25, 26, 27, revealing a potential role of Runx2 in vascular calcification. Pathogen‐induced inflammatory factors play a pivotal role in atherosclerotic arteries, potentially enhancing the differentiation and mineralization of vascular cells. Furthermore, extracellular‐regulated kinase (ERK) signaling is an important pathway to identify promising targets for therapeutic intervention in inflammatory diseases 28. ERK activation increases Runx2 transcriptional activity 29. The ERK pathway is an important connection between the cell surface and nucleus to control diverse cellular functions, including proliferation, differentiation, migration, and survival 30. The level of Runx2 is a decisive factor in the occurrence of osteoblastic differentiation 31. Our previous study demonstrated that OMV could induce oxidative stress activation through ERK1/2 9. However, the contribution of inflammatory factors derived from oral pathogenic bacteria to vascular calcification remains to be determined.

In the current report, we hypothesized that *P. gingivalis*‐derived OMV would affect VSMC differentiation and mineralization. Using an *in vitro* and *ex vivo* approach, we find that activation of Runx2 is necessary for OMV‐induced VSMC calcification. These studies demonstrate for the first time that periodontal pathogens may play a pivotal role in vascular calcification.

## Materials and methods

### Materials

Cell culture media were purchased from Invitrogen Technologies (Carlsbad, CA, USA). Real‐time PCR primers were purchased from Sangon Biotech (Shanghai, China). The miScript SYBR Green PCR kit was purchased from QIAGEN (Hilden, Germany). The Alkaline Phosphatase Colorimetric Assay Kit was purchased from Biovision (Milpitas, CA, USA). The Calcio Arsenazo III Proced was purchased from STANBIO laboratory (Boerne, TX, USA). The Pierce ECL Western Blotting KIT was purchased from Sigma‐Aldrich (St. Louis, MO, USA). Specific antibodies against Runx2, SM22α, α‐SMA, and OC were purchased from Cell Signaling Technology (Danvers, MA, USA). Antibodies against phosphorylated ERK1/2 and total ERK were obtained from Santa Cruz Technology (Santa Cruz, CA, USA). Specific β‐actin antibody was purchased from Sigma‐Aldrich. All secondary antibodies were purchased from Thermo Scientific (Waltham, MA, USA).

### Preparation of *P. gingivalis* OMV

Preparation of OMV was performed from culture supernatants of *P. gingivalis ATCC 33277* as previously described 6, 32, 33. In brief, the supernatant of a 2‐day culture of standard strain 33277 was collected by centrifugation at 4000 ***g*** for 20 min at 4 °C, then filtered through a 0.22‐μm polyvinylidene difluoride (PVDF) filter and ultracentrifuged at 150 000 ***g*** for 3 h at 4 °C in a 40 Ti rotor. The resulting OMV pellet was resuspended and the protein concentration was tested by the Bradford assay 34 using BSA as a criterion. Purified OMV was washed twice in 0.22‐μm filtered 0.01M PBS at 150 000 ***g*** for 2 h at 4 °C. OMV was isolated multiple times following the same protocol throughout the whole experiment. To avoid protein degradation, OMV was stored at −20 °C for short‐term storage (< 7 days) and −80 °C for long‐term storage (< 30 days). Transmission electron microscopy (TEM) was performed using a FEI Philips CM200EFG instrument (FEI Company, Eindhoven, The Netherlands) as previously described 35.

### VSMC isolation and cell culture

Primary VSMCs were isolated from the aortas of C57BL/6 mice (Sichuan University, Chengdu, China) as described previously 36, 37 and confirmed to express smooth muscle‐specific β‐actin antibody (β‐SMA) (Sigma‐Aldrich) by flow cytometry. All experiments were performed using VSMCs at passages 3–5.

Animal welfare and experimental procedures were implemented in line with the Guide for the Care and Use of Laboratory Animals (Ministry of Science and Technology of China, 2006). The Animal Ethics Committee of Beijing Institute of Translational Medicine authorized the study.

### *In vitro* VSMC calcification

Vascular smooth muscle cell calcification was induced by the addition of OMVs (0.1, 1 or 10 ng·mL^−1^) in osteogenic base media comprising 0.25 mm L‐ascorbic acid, 10 mm β‐glycerophosphate and 10^−8^ m dexamethasone (Gibco, Grand Island, NY, USA) for 3 weeks. Media changes were carried out every 3 days. Commercially available ultrapure *P. gingivalis* lipopolysaccharide (LPS) (Invivogene, San Diego, CA, USA) was used at a concentration of 10 ng·mL^−1^ as a comparison. Mineralization was determined by Alizarin Red staining (Sigma‐Aldrich).

### Analysis of cell viability

The 3‐(4,5‐dimethylthiazol‐2‐yl)‐2,5‐diphenyltetrazolium bromide (MTT) assay was used for determining cell viability and proliferation. In experiments, 2 × 10^4^ cells were added to each well on 24‐well gelatin‐coated tissue culture plates and stimulated with different concentrations of *P. gingivalis* OMV or LPS. After incubation for 4, 24, 48, and 72 h at 37 °C, 0.5 mg·mL^−1^ MTT (Sigma) in PBS was added to each well. The plates were incubated for 4 h at 37 °C, then the culture medium was removed and 500 μL of DMSO was added to each well followed by 5 min of incubation on a shaker. Optical density (OD) of each well was detected at 450 nm using a UV‐1200 spectrophotometer (Mapada, Shanghai, China). Cell viability data were expressed as percentages of the control.

### Total calcium measurement

Cells were dissolved in 0.5 mol·L^−1^ hydrochloric acid by shaking overnight at 4 °C. Total calcium in the cell lysates was determined by the Arsenazo III method according to the manufacturer's instructions.

### Real‐time polymerase chain reaction analysis

The effect of OMV on the expression of VSMC markers was determined by real‐time PCR. Total RNAs were isolated from VSMCs using Trizol reagent and reverse‐transcribed into cDNA. SYBR Green‐based real‐time PCR reactions were performed using specific primers for murine ALP 38, ColIA1 16, OC 17, Runx2 20, SM22α 21, α‐SMA 21,ERK1/2 29, and β‐actin as the control 38. Quantitative real‐time PCR was performed with the miScript SYBR Green PCR Kit on an iCycler Thermal Cycler (Bio‐Rad, Hercules, CA, USA) according to the manufacturer's instructions.

### Western blot analysis

Cells were treated with 10 ng·mL^−1^ OMV for 3 weeks and suspended in radioimmunoprecipitation (RIPA) lysis buffer. Proteins were separated and the preparation was transferred. Protein concentration was measured according to the protocol 37, 39. PVDF membranes were incubated overnight with primary antibodies for Runx2, ALP, ColIA1, OC (1/1000 dilution), SM22α,α‐SMA (1/2000), phosphorylated ERK1/2, total ERK (1/1000),β‐actin (1/6000). Secondary antibodies (1/10 000 for target proteins and 1/20 000 for β‐actin) were added for 1 h at room temperature. Protein bands were quantified using enhanced chemiluminescence ECL reagent (GE Healthcare, Little Chalfont, Buckinghamshire, UK).

### Detection of Runx2 Transactivity

To determine the transcriptional activity of the Runx2 protein, VSMCs were transfected with p6xRunxLuc luciferase reporter plasmid (0.5 μg) and SV containing a cDNA for Renilla reniformis luciferase (0.5 μg) using FuGENE 6. A minimal SV40 promoter was used to encode the *Renilla* luciferase gene downstream. VSMCs were washed after 24 h and treated with 10 ng·mL^−1^ OMV for another 48 h. The transfection efficiency of the luciferase reporter was normalized by Renilla Luciferase Activity System (Promega, Madison, WI, USA).

### Aortic ring culture and calcification

Under anesthesia, the aortas were instantly removed from 2‐month‐old C57BL/6 mice (25–30 g) prior to euthanasia. The vasculatures were placed into sterile dishes containing PBS, 1 μg·mL^−1^ penicillin, and 100 μg·mL^−1^ streptomycin (Pen‐Strep, Thermo Scientific), and refrigerated at 4 °C for 1 h. Under aseptic conditions, the aortas were dissected under a microscope and washed five times in PBS: Pen‐Strep (the same concentration as above) to remove all visible residuals. The aortas were cut into rings of about 3 ± 1 mm. Individual rings were placed into wells of sterile 12‐well plates (Costar, Sigma‐Aldrich) containing 1 mL Dulbecco's Modified Eagle's Medium (DMEM) with 1 mm sodium pyruvate/phenol red, Pen‐Strep, 2 mm glutamine, and 10% fetal calf serum (FCS). All aortic samples were placed at room temperature in an atmosphere of 5% CO_2_. Vessels were equilibrated at least 1 h before replacing the medium with fresh medium and the addition of stimulants. Vessels were incubated with PBS (control), OMV (10 ng·mL^−1^), or OMV cotreated with ERK inhibitor (20 μm) for 3 weeks with media/stimulator changes every 3 days. At 3 weeks, the vessels were embedded in paraffin and cut into 8‐μm serial sections. Frozen aortic sections were stained using silver nitrate by Von Kossa (Sigma‐Aldrich) to detect calcium deposits. Stained specimens were examined under a light microscope (DM3000, Lecia, Wetzlar, Germany).

### Statistical analysis

Quantitative data are expressed as means ± SD of at least three separate experiments. Statistical significance was determined using one‐way ANOVA and significance was established at a *P* value < 0.05.

## Results

### *Porphyromonas gingivalis* OMV induces VSMC calcification in a concentration‐dependent manner

**Figure 1:**
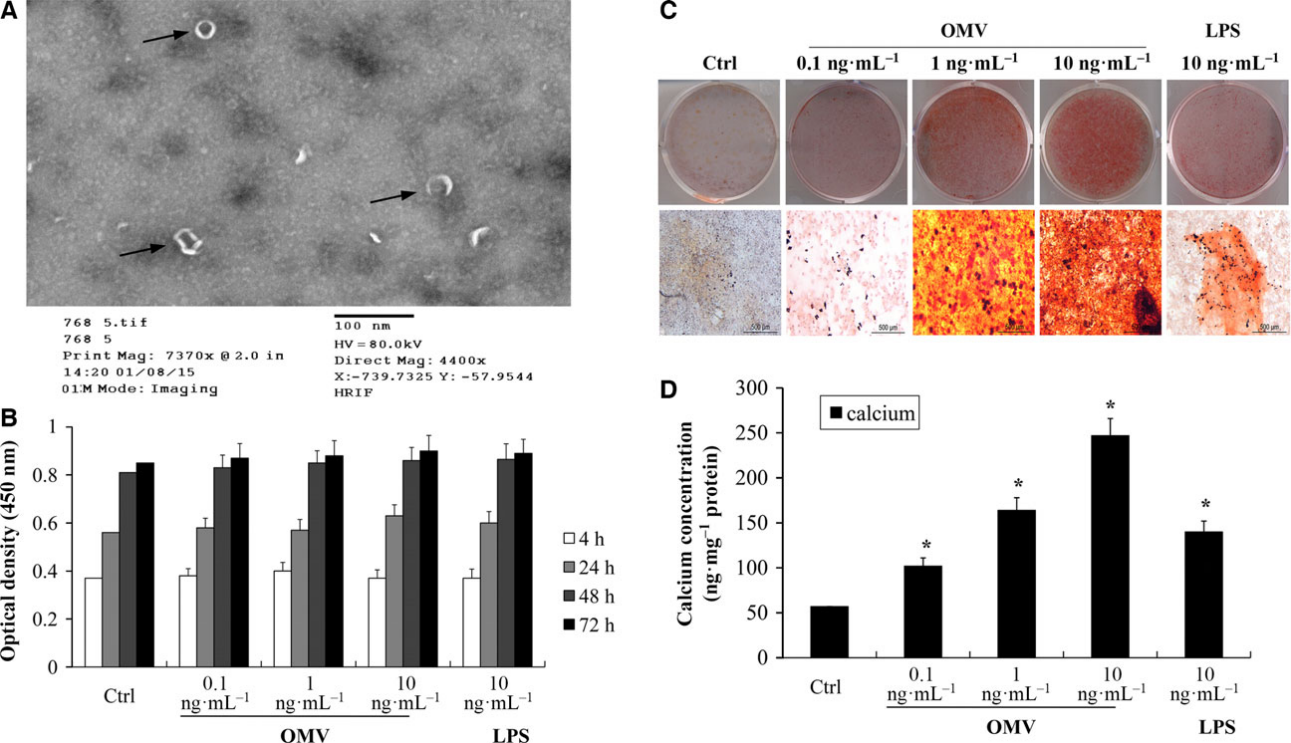
*Porphyromonas gingivalis* OMV induces VSMC calcification. (A) Image of purified OMV identification through TEM. (B) The cell viability was measured after 4, 24, 48, and 72 h using the MTT assay. The *y*‐axis represents mean ± SD values of optical densities measured at 450 nm from four wells of one representative experiment. (C) VSMCs were exposed to 0.1, 1, and 10 ng·mL^−1^ OMV and 10 ng·mL^−1^ LPS in osteogenic media for 3 weeks with changing media every 3 days. VSMC calcification was determined by Alizarin Red staining. Representative images of stained dishes (upper) and microscopic views (×40, lower) are shown. (D) Total calcium levels were determined by the Arsenazo III method according to the manufacturer's instructions. The results from three independent experiments performed in duplicate are shown (*P < 0.001, compared with control).

Outer membrane vesicle purity and structure were determined using TME. The purified OMV presented with small spherical structures in the absence of periplasmic components such as cytoplasmic, inner membrane proteins, and cellular fragments. *Porphyromonas gingivalis* OMV was found to be spherical in shape and have a size distribution of 80–150 nm, as determined by TEM (Fig. 1A). We then examined the direct effect of OMV or LPS on VSMC calcification *in vitro*. We found that OMV at concentration above 50 ng·mL^−1^ led to cell death within 17 days (data not shown). OMV at nontoxic concentrations of 0.1–10 ng·mL^−1^ induced VSMC calcification in a concentration‐dependent manner, presented as an MMT assay histogram (Fig. 1B), Alizarin Red staining (Fig. 1C) and total calcium levels (Fig. 1D). No significant change in cell viability was observed after treatment with the lower concentrations of OMV (0.1–10 ng·mL^−1^) even after 3 days. In addition, the presence of 10 ng·mL^−1^ LPS caused a mild effect toward calcifying VSMCs. OMV‐induced calcium levels were much higher than those of LPS activation at the same concentration. Taken together, we established a reproducible *P. gingivalis* OMV‐induced differentiation and calcification model of VSMCs *in vitro*.

### OMV induces the expression of bone markers and down‐regulates SMC markers

**Figure 2:**
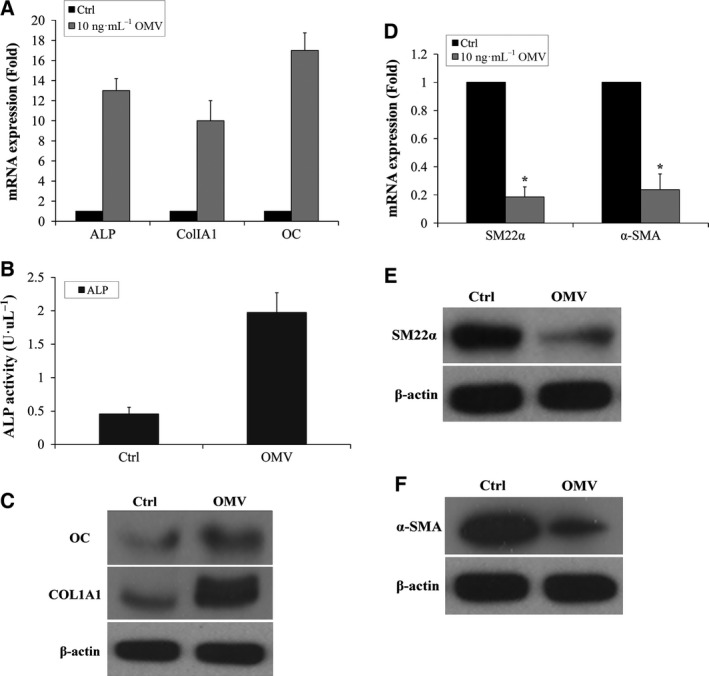
OMV induces the expression of bone markers and down‐regulates SMC Markers. (A) Expression of bone markers ALP, ColIA1, and OC was determined by RT‐PCR during VSMC calcification. VSMCs were exposed to 10 ng·mL^−1^ OMV in osteogenic media for 2 weeks. (B) The Alkaline Phosphatase Colorimetric Assay was performed in VSMCs exposed to 10 ng·mL^−1^ OMV for 15 days. (C) Expression of ColIA1 and OC protein was determined by western blot analysis. (D) Expression of SMC markers SM22α and α‐SMA was determined by RT‐PCR in VSMCs exposed to 10 ng·mL^−1^ OMV in osteogenic media for 2 weeks. (E) Expression of SMC marker SM22α protein. (F) Expression of SMC marker α‐SMA protein. Results from three independent experiments performed in duplicate are shown (*P < 0.001, compared with control).

Accumulating evidence indicates that vascular calcification resembles the process of osteogenesis 40, 41. We therefore evaluated the effect of OMV on osteogenic differentiation of VSMCs and measured the expression of bone and SMC markers by real‐time PCR and western blot. Expression of the bone markers ALP, ColIA1, and OC were increased in 10 ng·mL^−1^ OMV‐treated osteogenic media at 2 weeks (Fig. 2A,C) both at RNA and protein level. The Alkaline Phosphatase Colorimetric Assay showed increased expression of ALP activity following exposure to 10 ng·mL^−1^ OMV for 15 days (Fig. 2B). In contrast, there was a dramatic decline in SM22α and α‐SMA expression during VSMV osteogenic differentiation (Fig. 2D–F). These data demonstrate that induction of bone markers and suppression of SMC‐specific phenotype are associated with OMV‐induced VSMC calcification.

### OMV induces Runx2 expression and transactivity in VSMCs

**Figure 3:**
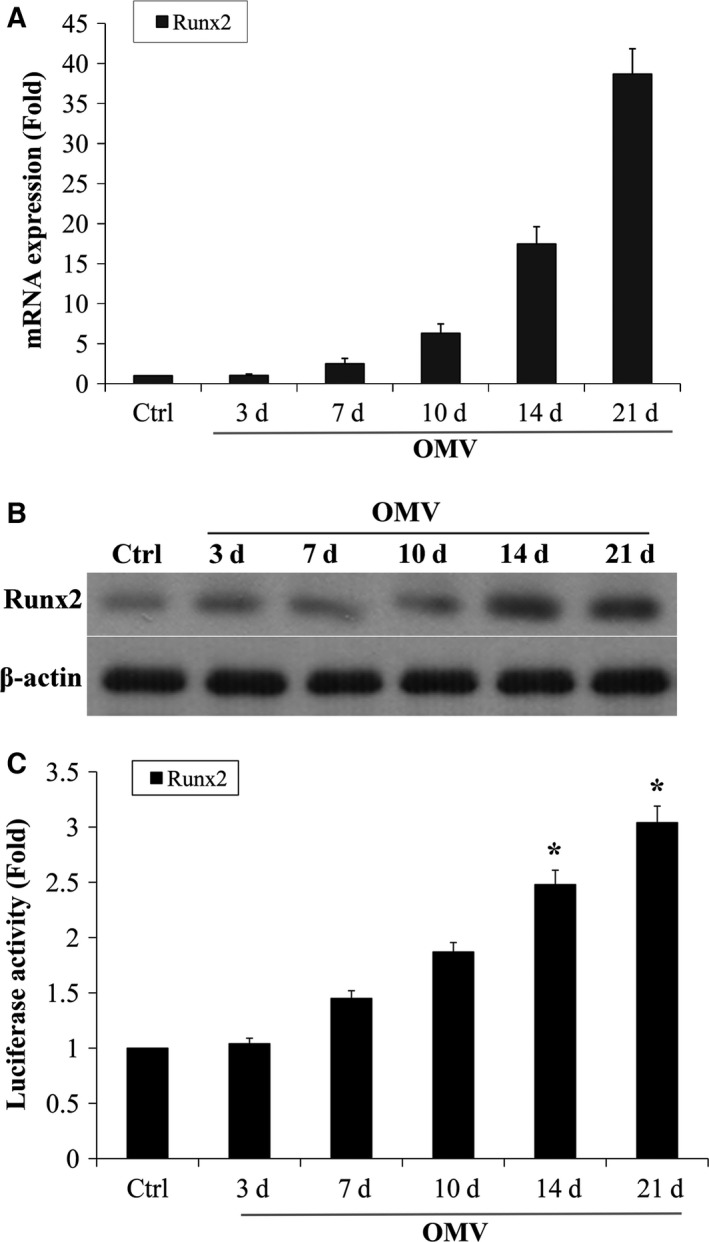
OMV induces Runx2 expression and transactivity in VSMCs. (A) Expression of Runx2 in VSMCs exposed to 10 ng·mL^−1^ OMV in osteogenic media for 3–21 days was determined by RT‐PCR. Results from three independent experiments performed in duplicate are shown (*P < 0.001, compared with control). (B) Expression of Runx2 protein was determined by western blot analysis. (C) Runx2 transactivity in VSMCs was determined with the use of p6xRunxLuc and a Renilla luciferase expression plasmid. The transfection efficiency of the luciferase receptor was normalized by Renilla Luciferase Activity System. Results from three independent experiments performed in duplicate are shown (*P < 0.001, compared with control).

Runx2 is a key transcription factor that regulates osteoblast differentiation and has been shown to induce ALP activity and regulate expression of many bone martric markers 23. We therefore determined the potential effect of OMV on the expression and transactivity of Runx2 in VSMCs. Quantitative real‐time PCR confirmed an increase in Runx2 mRNA when VSMCs were treated with 10 ng·mL^−1^ OMV for 3–21 days (Fig. 3A). Furthermore, western blot analysis confirmed that the OMV‐induced increase in Runx2 mRNA translated to protein expression (Fig. 3B). Runx2 protein expression was increased in VSMCs exposed to OMV in a time‐dependent manner. To determine the transcriptional activity of the Runx2 protein, a luciferase receptor assay was performed. A multimeric Runx2 promotor receptor was transfected into VSMCs and then the cells were induced by OMV (Fig. 3C). The data suggest that OMV induces Runx2 expression and transactivity in VSMCs, which may contribute to cellular calcification.

### ERK signaling mediates Runx2 activation and VSMC calcification

**Figure 4:**
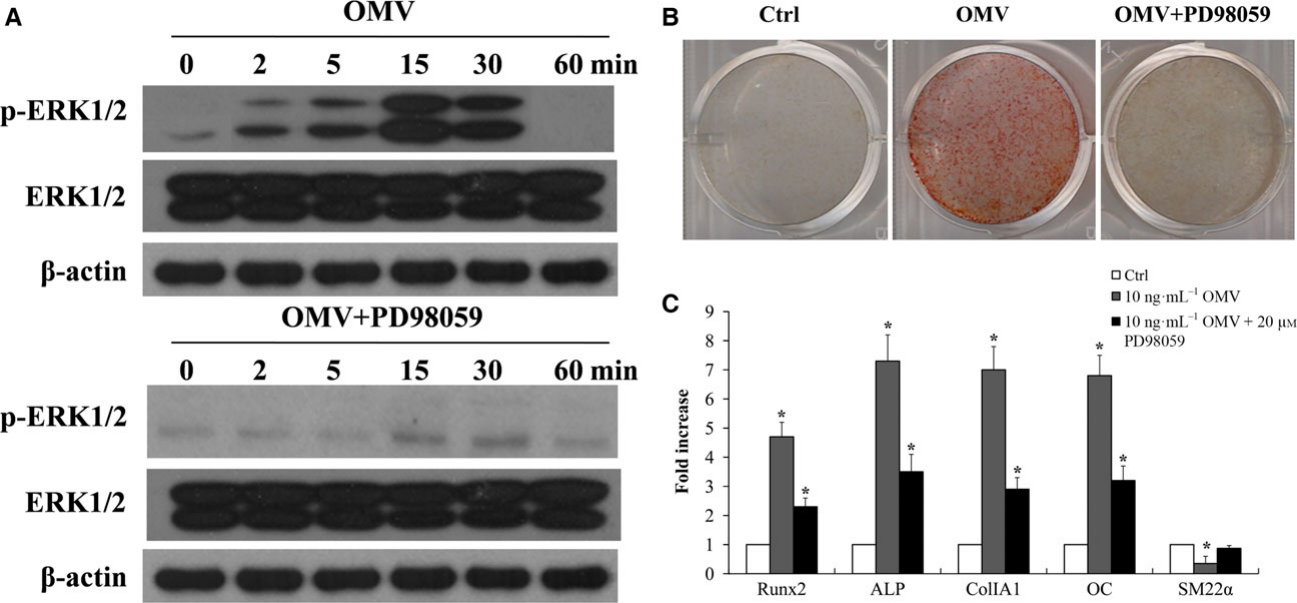
ERK signaling mediates Runx2 activation and VSMC calcification. (A) Expression of phosphorylation ERK1/2 was determined in VSMCs exposed to 10 ng·mL^−1^ OMV in osteogenic media by western blot analysis. Representative results from three independent experiments in duplicate are shown (upper). VSMCs were pretreated with ERK1/2 inhibitor (PD98059) 20 μm for 30 min and then exposed to 10 ng·mL^−1^ OMV in osteogenic media. The treatment was repeated every 3 days, and cells were treated for 2 weeks. Expression of p‐ERK1/2 was determined in VSMCs exposed to 10 ng·mL^−1^ OMV cotreated with PD98059 by western blot analysis (lower). (B) VSMC calcification was determined by Alizarin Red staining. (C) Effect of MAPK inhibitor on the expression of Runx2, ALP, ColIA1, OC, and SM22α was determined by RT‐PCR. VSMCs were pretreated with PD98059 (20 μm) for 30 min and then exposed to 10 ng·mL^−1^ OMV for 2 weeks in osteogenic media. The results are presented as the fold increase relative to the control group (mean ± SD,* n* = 3). Representative results from five independent experiments in duplicate are shown (*P < 0.01, compared with control).

Extracellular‐regulated kinase is an important member of the mitogen‐activated protein kinase (MAPK) family and is activated by oxidative stimulation with H_2_O_2_ or OMV in a variety of cells 16, 42, 43. We therefore investigated whether activation of phosphorylated ERK1/2 by OMV was required for VSMC calcification. We determined that OMV induced rapid activation of ERK1/2, an important mediator for intracellular calcium transduction (Fig. 4A). Moreover, pharmacological inhibition of ERK1/2 by PD98059 (20 μm) prevented OMV‐induced VSMC calcification (Fig. 4B). Furthermore, the increased expression of Runx2, ALP, ColIA1, and OC was significantly interdicted by MAPK inhibition, while OMV‐induced down‐regulation of SMA22α was not restored (Fig. 4C). Our data indicate that ERK1/2 is able to participate in the up‐regulation of Runx2 and VSMC calcification.

### Representative immunohistochemistry image of VSMC calcification in mice aortas

**Figure 5:**
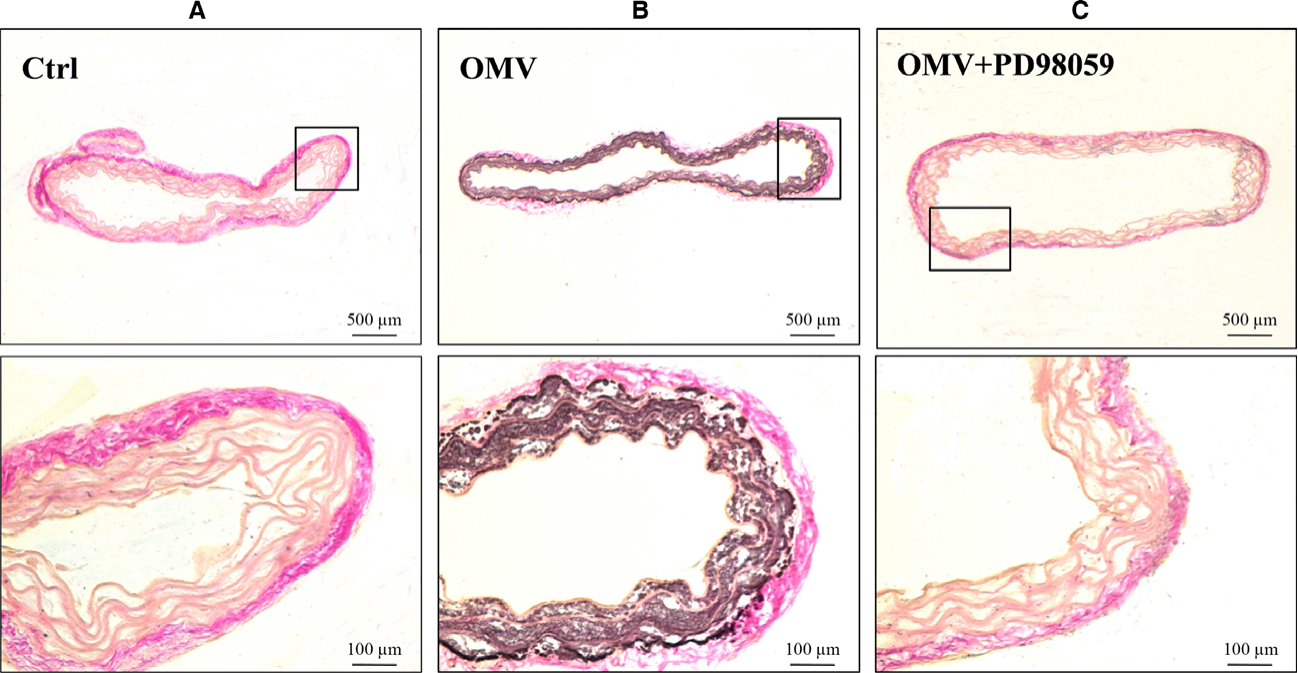
Immuohistochemistry for OMV‐induced VSMC calcification in mouse aorta. (A) After 3 weeks of aorta ring culture with PBS, aortic sections were stained using silver nitrate to detect calcium deposits. (B) Aorta rings cultured in the stimulation of 10 ng·mL^−1^ OMV for 3 weeks. (C) Aorta rings were cotreated with 10 ng·mL^−1^ OMV and ERK inhibitor. Representative images from three independent experiments are performed in duplicate. Higher magnification images of the boxed areas are shown under each image.

For further study into the effect of ERK1/2 on OMV‐induced Runx2 activation and VSMC calcification, *ex vivo* experimentation was performed. An aorta ring culture model was used to detect calcium deposits in VSMCs following Von Kossa staining. Aorta rings were cotreated with ERK1/2 inhibitor and OMV for 3 weeks. The results showed that OMV stimulated vascular calcification compared with the control, and this effect was blocked when PD98059 was added (Fig. 5A–C). This result further confirmed the activation role of ERK1/2 on the OMV‐induced expression of Runx2 up‐regulation and VSMC calcification. This data demonstrates that Runx2 is an essential component of OMV‐induced calcification of VSMCs, and ERK signaling plays a vital role in mediating Runx2 up‐regulation and VSMC calcification.

## Discussion

Epidemiological links between periodontitis and angiocardiopathy predict that a direct correlation exists between these two diseases. One of the rationales for this mechanistic link is that periodontitis patients have frequent bacteremic episodes, with LPS, gingipain, and other virulence factors of periodontal disease often detected in their circulation. The presence of serum lipids (and their modification), engagement of receptors on inflammatory cytokines and cells, invasion of endothelial cells, and diffusion of atheromatous lesions could therefore be attributed to bacteria and their components that could further promote inflammatory responses, thus contributing to atherosclerotic plaque formation. OMV retains virulence and can be internalized by host cells or released into the circulation. In the sera of periodontitis patients, significantly stronger reactivity was observed on the OMV‐induced wild‐type strain than the isogenic OMV‐depleted strain 5. One possible hypothesis is that OMV may exert its function by serving as a transport vehicle for toxin delivery.

To the best of our knowledge, there are no published data illustrating the causal association between periodontitis and vascular calcification. First, we thus evaluated the effects of OMV or LPS on VSMC calcification, which indicated that OMV induced osteogenic differentiation and calcification of VSMCs in a dose‐dependent manner at a non‐toxic concentration (0.1–10 ng·mL^−1^). This result was further confirmed by measuring total production of calcium, the deposition of which in the vessel wall is a characteristic of advanced atherosclerosis, with reduced elasticity and compliance of the vessel wall 1. Our result identified a positive connection between *P. gingivalis*‐derived OMV and VSMC calcification. Many studies indicate that *P. gingivalis* LPS plays a critical role in the pathogenesis of cardiovascular disease. Interestingly, LPS‐only induction plays a relatively minor role in stimulating vascular cellular calcification, indicating that other virulence components of OMV might be involved in the calcification process. Here, we focused on the impact of OMV on VSMC calcification and found that OMV‐induced VSMC calcification was relevant to down‐regulation of VSMC‐specific markers and increased expression of bone markers, indicating a phenotypic change of VSMCs into osteoblast‐like cells.

More importantly, we demonstrated that VSMC differentiation following stimulation with OMV is mediated by Runx2, a key transcription factor for osteoblast and chondrocyte differentiation 19, 20, 27, 39, 44. ERK1/2 plays a pivotal role in Runx2 activation and subsequently accelerates VSMC calcification 16, 42, 45. *Porphyromonas gingivalis*‐derived OMV has the ability to activate the MAPK pathway 9, which may lead to activation of Runx2. We found that OMV‐activated ERK signaling was required for OMV‐induced calcification of VMSCs. In contrast, pharmacological inhibition of MAPK by PD98059 46 reduced the expression and transactivity of Runx2, as well as the expression of bone markers during VSMC calcification. These results are consistent with activated intracellular signaling increases in gene and transcriptional expression of Runx2 during osteoblast and chondrocyte differentiation 47. Our data provide further support that Runx2 is an essential component of OMV‐induced calcification of VSMCs and suggests that ERK1/2 is able to participate in the up‐regulation of Runx2 and VSMC calcification.

In summary, our results reveal an activation role of *P. gingivalis*‐derived OMV in mediating VSMC calcification both *in vitro* and *ex vivo*. Runx2, which is affected by upstream of ERK signaling, exerts a great influence on OMV‐induced VSMC differentiation and calcification. Our findings provide vital molecular insight into the effect of ERK1/2‐Runx2 on vascular calcification and a possible causal relationship between periodontitis and vascular calcification.

## Author contributions

YJ designed the experiment. WWY and BG completed the project and acquired the data. WYJ helped with the animal experiment. YJ and WWY interpreted the data and wrote the paper. WWY and BG contributed equally to this work and are joint first authors.
